# Knockdown of LMNB1 Inhibits the Proliferation of Lung Adenocarcinoma Cells by Inducing DNA Damage and Cell Senescence

**DOI:** 10.3389/fonc.2022.913740

**Published:** 2022-05-31

**Authors:** Jiangbo Li, Zhijia Sun, Yingshu Cui, Lingmei Qin, Fengyun Wu, Yufang Li, Nan Du, Xiaosong Li

**Affiliations:** ^1^ Department of Biology, Beijing Institute of Biotechnology, Beijing, China; ^2^ Medical School of Chinese People's Liberation Army (PLA), Chinese People's Liberation Army General Hospital, Beijing, China; ^3^ College of Life Sciences, Capital Normal University, Beijing, China; ^4^ Department of Oncology, Fourth Medical Center of Chinese PLA General Hospital, Chinese People's Liberation Army General Hospital, Beijing, China; ^5^ Department of Oncology, Seventh Medical Center of Chinese PLA General Hospital, Chinese People's Liberation Army General Hospital, Beijing, China

**Keywords:** LMNB1, LUAD, chromosome accessibility, telomere, cell senescence

## Abstract

**Background:**

Lung cancer has considerably high mortality and morbidity rate. Lung adenocarcinoma (LUAD) tissues highly express lamin B1 (LMNB1), compared with normal tissues. In this study, we knocked down LMNB1 in LUAD cells A549 and NCI-1299 to explore the effect of its inhibition on the proliferation of cells and the potential mechanism.

**Methods:**

Using bioinformatics methods, we analyzed the specificity of LMNB1 mRNA expression level in LUAD and its effect on prognosis from TCGA data. SiRNAs were used to knock down LMNB1 in the A549 cell line, and the knockdown effect was identified by western blotting and qRT-PCR. Through CCK8 cell proliferation assay, wound healing assay, TRAP, cloning formation Assay, DNase I-TUNEL assay, ATAC-seq, immunofluorescence, FISH, *in vivo* mouse xenograft studies, etc, we evaluated the influence and mechanism of LMNB1 on LUAD cell line proliferation *in vitro* and *in vivo*.

**Results:**

According to bioinformatics analysis, LMNB1 is substantially abundant in LUAD tissues and is associated with tumor stage and patient survival (P < 0.05). After silencing LMNB1, the rate of cell growth, wound healing, the number of transwells, and the number of cell colonies all decreased significantly (P < 0.01). With the decreased LMNB1 expression, H3K9me3 protein expression decreases, chromosome accessibility increases, P53, P21, P16 and γ-H2AX protein expression increases, and the number of senescence staining positive cells increases. At the same time, *in vivo* mouse xenograft experiments showed that the tumor volume of the LMNB1-silenced group was significantly reduced, compared to that of the control group (P < 0.01), and the proliferation biomarker Ki-67 level (P < 0.01) was considerably reduced.

**Conclusions:**

Overexpression of LMNB1 in LUAD cells is significant, which has excellent potential to be an indicator for evaluating the clinical prognosis of LUAD patients and a target for precise treatment.

## Background

Lung cancer is a major human cancer with high morbidity and mortality (1–3). It can be categorized into small cell lung cancer (SCLC) and non-small cell lung cancer (NSCLC). Among these, NSCLC accounts for 85-90% of lung cancer, and lung adenocarcinoma (LUAD) is the most common type of NSCLC, accounting for almost half of the lung cancers (4, 5). LUAD is generally accepted to develop from atypical adenomatous hyperplasia to adenocarcinoma in situ, then to micro-invasive adenocarcinoma, and finally to overt invasive lung adenocarcinoma (6). Although LUAD generally grows slower and has smaller masses than the squamous lung cancer during the same period, LUAD tends to start metastasizing early (7). In addition, there is a lack of clinically sensitive and specific screening biomarkers, and the clinical symptoms in the early stage of onset are not typical. Most of the patients diagnosed have developed into late-stage or metastatic LUAD (8, 9).

The nucleus is considered one of the most important organelles in eukaryotic cells as it houses genome that contains the entire cellular genetic map (10). The nuclear fiber layer, which is found in the inner layer of the nuclear envelope, is primarily a protein network with many proteins, including lamins, which play a role in maintaining the physiological balance of the cells (11). Lamins are of two types - type A and type B. Type A includes lamin A and lamin C. When the cell lacks A-type nuclear laminins, the cell’s viability and the expression of mechanically sensitive genes is reduced, changing the response of the nuclear structure to mechanical distortion. Type A lamins occupy a vital position in the process of force transmission (12, 13). LMNB1, LMNB2, and LMNB3 are all members of type B, with the LMNB1 gene encoding Lamin B1. LMNB1 is a critical nuclear skeleton component protein (14). In recent years, research on the activity of B-type proteins, particularly LMNB1, has received an increasing amount of attention, particularly in LUAD (15, 16). Through biometric analysis, we discovered that LMNB1 is substantially abundant in LUAD and is associated with cancer stage and patient prognosis in this study. The A549 cell line’s proliferation and migration were significantly inhibited when LMNB1 was knocked out. The high expression of P53, P21, P16 protein and the increase of senescence staining positive cells indicated that the tumor cells had obvious senescence, and the expression of H3K9me3 protein decreased, and γ- Elevated H2AX expression and DNase I-Tunel experiment showed that the accessibility of cell chromosomes increased and DNA damage occurred. In addition, mouse tumor formation experiments confirmed the effect of LMNB1 on tumor growth. Therefore, our findings confirm that LMNB1 is an essential molecule in the incidence and progression of LUAD, and targeting LMNB1 can be an important strategy for treating LUAD.

## Methods

### Cell Culture and Reagents

The 293T human embryonic kidney cell line, A549 and NCI-1299 cell lines were purchased from ATCC (The American Type Culture Collection, USA) and regularly tested for mycoplasma infection. Dulbecco’s Modified Eagle Medium (DMEM) supplemented with 10% fetal bovine serum (Hyclone, China) was regularly used to cultivate the cells. Cells were cultured in dedicated cell culture incubators that were maintained at 37°C with 5% CO_2_.

### RNA Interference

siRNAs for LMNB1 and corresponding negative controls (si NC) were purchased from Invitrogen (USA). According to the manufacturer guidelines, we used RNAiMax (Invitrogen, USA) for transfections. The target sequences are as follows.

LMNB1 siRNA#1: 5’-GAAGGAAUCUGAUCUUAAU-3’;
LMNB1 siRNA#2: 5’-GAAAGAGUCUAGAGCAUGU-3’.
CBX5 siRNA#1: 5’-CCCAGGGAGAAGUCAGAAATT-3′;
CBX5 siRNA#2: 5’- GGCAAGTGGAATATCTGTTGA-3′.
SUV39H1 siRNA#1: Forward: GAGUACCUGUGCGAUUACATT, Reverse: UGUAAUCGCACAGGUACUCTT;
SUV39H1 siRNA#2: Forward: CCUUCGUGUACAUCAAUGATT, Reverse: UCAUUGAUGUACACGAAGGTT.

Lentiviral shRNA vectors of LMNB1 were constructed by cloning short hairpin RNA fragments into pSIH-H1-Puro (System Biosciences, USA). Co-transfection of HEK293T cells with recombinant lentivirus vectors and pPACK Packaging Plasmid Mix (System Biosciences, USA) in the presence of PEI reagent (Polyscience, USA) resulted in the generation of lentiviruses. Lentiviruses were used to infect target cells according to the manufacturer’s guidelines.

### Western Blotting

Western blot analysis was performed according to the standard procedure. Anti-Lamin B1 (66095), β-actin (01003), P16 (10883), and P21 (10355) were purchased from proteintech (Wuhan, Hubei, China). Anti-Lamin A/C (SAB4200236) was purchased from sigma (Shanghai, China). Both γ-H2AX (ab2893) and H3K9me3 (ab8898) were purchased from abcam (Shanghai, China). P53 (9284S) was purchased from CST (Shanghai, China). SDS–polyacrylamide gels were used to separate the samples, and then tansfers were done onto nitrocellulose (NC) membranes. Corresponding antibodies were used to incubate the membranes. ECL Luminous Liquid (Millipore, China) was used to develop the target protein band and the images were analyzed using Image LabTM software (Bio-Rad, China).

### Assays for CCK8 Cell Proliferation and Wound Healing

The A549 cells with knocked down LMNB1 were seeded onto 96-well plates (3 × 10^3^ cells per well plate). CCK8 was used to monitor cell proliferation following the manufacturer’s recommendations (Dojindo biochemical techniques). In the ultra-clean workbench, a horizontal lines were drawn on the back of the 6-well plate with a thin marker pen, each spacing 1 cm across the plate hole. The A549 cells transfected with siLMNB1 and the negative control group were spread on a 6-well plate. A total of 2 ml of medium was added to each hole to culture for 20 h, allowing growth to form single-layer cells. A 10 μl pipette was used to draw the vertical, horizontal line. PBS was slowly added to the wall in each pore cell to wash two times to remove the damaged cells. 2 ml of serum-free medium was gently added along the pore wall to avoid dispersing single-layer wall cells. 6-well plate was moved to the incubator to continue the culture and continuos pictures were taken using a microscope after 0 hours, 6 hours, 12 hours and 24 hours.

### Transwell Assays

Cells were collected and suspended in serum-free medium. Six hundred microliters of medium containing 20% FBS was added to the 24-well plate, and about 200 μl cell suspension was added to the upper chamber with Matrigel, and cultured for 16 h. The chamber was fixed in 4% paraformaldehyde and then stained in 0.1% crystal violet solution for 30 minutes, respectively. A cotton swab was used to wipe the cells and matrigel out of the chamber. The images were observed under a microscope, and the number of invasive cells was calculated using the Image J software.

### Assay for Cloning Formation

Cells were harvested during their log phase and seeded at a density of 500, 1000, or 1500 cells per well in a six-well plate. After a two-week culture period, cells were fixed with 4% paraformaldehyde and stained with crystal violet (Solarbio, C8470, China).

### DNase I-TUNEL Assay

The cell chromosome accessibility experiment was carried out as described earlier (17) and modified appropriately. The general steps are as follows: cells were permeabilized in extraction buffer (50 mM NaCl; 3 mM MgCl2; 0.5% Tritionx-100; 300 mM Sucrose dissolved in 25 mM HEPES, pH7.4) for 15 min before digesting with 0 U, 0.03 U, 0.1 U of DNase I (NEB) respectively. Cells were then fixed in 4% PFA. TUNEL tests (DeadEnd™ Fluorometric TUNEL System, Promega) were subsequently performed, as per the manufacturer’s recommendations. The nuclear area was defined according to DAPI DNA staining. Confocal images were collected using a Radiance2100 confocal microscope (Bio-Rad).

### ATAC-Seq

A549 shLMNB cells and control group were sent to Beijing Novogene Co., Ltd. for ATAC-seq assay.

### TRAP Assay

The TRAP assay was carried out using the TRAPeze^®^ Telomerase Detection kit (Millipore, China). In brief, cultivated cell pellets were lysed for 30 minutes on ice with 1× CHAPS lysis buffer. Total protein concentration was measured using the Bio-Rad Protein Assay Kit after centrifugation at 12,000 rpm for 30 minutes. The specified amounts of samples were combined with 2 µl of TRAP buffer, and final reaction compounds were mixed with GelRed loading buffer (Generay Biotech, China) and run on 10% polyacrylamide gel (29:1 acryl/bisacryl) in 0.5×Tris-borate-EDTA (TBE). The gel was operated at 200V for 50 minutes at room temperature. ChemiDoc™ Imaging System (Bio-Rad) was used to photograph the gel.

### Immunofluorescence

50 ml PBS mixed with 500 μl NGS was prepared as a 1% blocking solution. 10 ml 1% blocking solution mixed with 50 μl TritonX-100 was prepared as 0.5% previous liquid. Cells grown in a petri dish were fixed using 4% PFA for fifteen minutes at room temperature, permeabilized using 0.5% previous liquid for twenty minutes on ice, rinsed three times with 1% blocking solution for 10 min. The petri dish was then incubated for two hours at room temperature with appropriate antibodies, rinsed three times with blocking solution for 10 minutes, then set in the dark for one hour with appropriate secondary antibodies. The cells were rinsed three times with PBS for ten minutes following incubation. DAPI was used to stain the nuclei. Confocal images were collected using a LSM 780 confocal microscope (Zeiss).

### PCR for Quantitative Reverse Transcription

TRIzol reagent was used to extract total RNA, and reverse-transcribed using SuperScript II Reverse Transcriptase according to the protocol (Invitrogen). On a CFX96 Real-Time PCR detection technique, real-time quantitative PCR was carried out using SYBR-green premix (Takara, China). The following sequences of primers were used for qRT-PCR.

LMNB1 forward: 5’-AAGCAGCTGGAGTGGTTGTT-3’;
LMNB1 reverse: 5’-TTGGATGCTCTTGGGGTTC-3’.
LMNA/C forward: 5’-ACGGCTCTCATCAACTCCACTG-3’;
LMNA/C reverse: 5’-TCCTCATCCTCGTCGTCCTCAA-3’.
p21 forward: 5’-TCGCTCAGGGGAGCAGGCTGAA-3’;
p21 reverse: 5’-CTCGCGCTTCCAGGACTGCAGGCT-3’.
p53 forward: 5’-AGAGTCTATAGGCCCACCCC-3’;
p53 reverse: 5’-GCTCGACGCTAGGATCTGAC-3’.
p16 forward: 5’-CTTCCTGGACACGCTGGT-3’;
p16 reverse: 5’-GGGATGTCTGAGGGACCTT-3’.
β-actin forward: 5’-ATCACCATTGGCAATGAGCG-3’;
β-actin reverse: 5’-TTGAAGGTAGTTTCGTGGAT-3’.

As an internal control, β-Actin was used. The comparative Ct technique was used to compute the relative expression.

### Assay for SA-β-Gal

SA-β-gal test was carried out using an Aging Staining Kit (Beyotime, China), according to the manufacturer’s protocol. After staining, the cells were observed under a fluorescence microscope. Five fields of vision were taken from each sample to observe the proportion of cells stained dark blue in each field.

### qPCR Assay for Average Telomere Measurement

Genomic DNA was isolated from the cells using KingFisher Flex DNA purification instrument (ThermoFisher, USA) with MagMAX™ DNA Multi-Sample Ultra 2.0 Kit (ThermoFisher, USA). The biomarkers for Telomere PCR were tel1b: 5-CGGTTT(GTTTGG)_5_GTT-3, used at a fixed concentration of 300 nM, and tel2b: 5-GGCTTG(CCTTAC)_5_CCT-3, used at a final volume of 300 nM. The probes for single-copy gene (36B4) PCR were 36B4u: 5-CAGCAAGTGGGAAGGTGTAATCC-3, used at a final concentration of 300 nM, and 36B4d: 5-CCCATTCTATCATCAACGGGTACAA-3, used at a desired volume of 500 nM. The 2×Mix (Qiagen, USA) was used in the qPCR reaction mixture with 9.2 ng genomic DNA in each tube. qPCR was carried out on CFX-96 qPCR instrument (Bio-Rad, USA). The Telomere (T) PCR settings were 95°C for 10 minutes, followed by 20 cycles at 95°C for 15 seconds and 56°C for 1 minute. The 36B4 (S) PCR conditions were 95°C for 10 minutes and 30 cycles at 95°C for 15 seconds and 60°C for 1 minute. The relative T/S ratio of each sample was calculated as the relative telomere length. The T/S ratio for each sample was measured twice.

### Animal Experiments

Protocols were agreed upon by the Institutional Animal Care and Use Committee of the Beijing Institute of Biotechnology. Eight four-week-old female BALB/c nude mice were randomly divided into two categories, with four mice in each group. One class was injected with A549 cells stably knockdown LMNB1, and the other class was injected with control A549 cells. A mixture of 100 μL of cell suspension (containing 5×10^6^ cells) and 100 μL of matrix glue was injected into the back of nude mice. Tumor growth was measured with a Vernier caliper after seven days, and the volume of tumors was determined using the following formula: volume = (longest diameter × shortest diameter^2^)/2. On day 30 following implantation, the mice were slaughtered. The weight of nude mice was measured, and subcutaneous tumors were dissected and isolated. Tumor size was measured. The tumor tissues were divided into 4 parts. The qRT-PCR was performed to determine the transcription of P53, P21 and P16 in the tissues. Secondly, paraffin-embedded tissues were sectioned. The paraffin slices were immunohistochemically examined for LMNB1 and Ki-67 expression. Q-FISH was examined for telomere length. The last tumor tissues were used in TRAP to detect telomerase activity.

### Quantitative Fluorescence *In-Situ* Hybridization

The paraffin slides containing tissue were incubated in 10 mM sodium citrate (pH 6.5) at 88°C for 10 minutes, rinsed with PBS(pH 7.2) at room temperature for 1 minute in a glass slide dyeing vat, dried with 25%, 50%, and 95% ethanol, and treated with 1% pepsin solution in a glass slide dyeing vat at 37°C for 2 minutes. Each slide was incubated with 80 μl 10 mg/ml RnaseA solution (NanoMagBio, Wuhan), covered with cover glass, and placed in the heating block at 37°C for 2 h. The cover glasses were removed, and the slides were rinsed with PBS for 1 minute. The slides were then briefly immersed in 25%, 50%, and 95% ethanol and air-dried. Next, each sample was hybridized with a 100 μl telomere probe (TelC-Alexa594, PANAGENE) overnight in the dark at room temperature (at least 16 hours). After hybridization, the slides were placed in a Coplin flask with 70% formamide buffer and rinsed for 15 minutes. Then the slides were rinsed with fresh formamide buffer four times, 15 minutes each time, followed by Tween 20 buffer four times for 5 minutes at room temperature. Nuclei were counterstained with 80 μl DAPI(dilution of 1:1000) for 5 minutes at room temperature. The slides were sealed with a 20-25 μl vent shield and observed under a fluorescence microscope as soon as possible or stored in a closed box at -20°C. Image J program was used to evaluate the fluorescence intensity of Telomere.

### Immunohistochemistry

Tissues from tumors were soaked in formalin before being embedded in paraffin. Tissue sections were 4 μm thick, and dewaxing, hydration, and antigen healing were performed as per standard procedures. 3% hydrogen peroxide inhibited catalase activity. The sections were then treated overnight at 4°C with LMNB1 and Ki-67 antibodies. They were then treated for 1 hour at 37°C with a secondary antibody (ZSGB-BIO, PV-6000, China). After DAB staining (Solarbio, DA1010, China), the nuclei were stained with hematoxylin (Solarbio, G1120, China).

### Statistical Analysis

Assessments between two groups were performed using Student’s *t-*test. The data are presented as the means ± standard deviation (SD); P < 0.05 was found to be significant. *NS* means statistically insignificant.

## Results

### Highly Expressed LMNB1 in LUAD Cells Is Associated With Tumor Stage and Overall Survival

**Figure 1:**
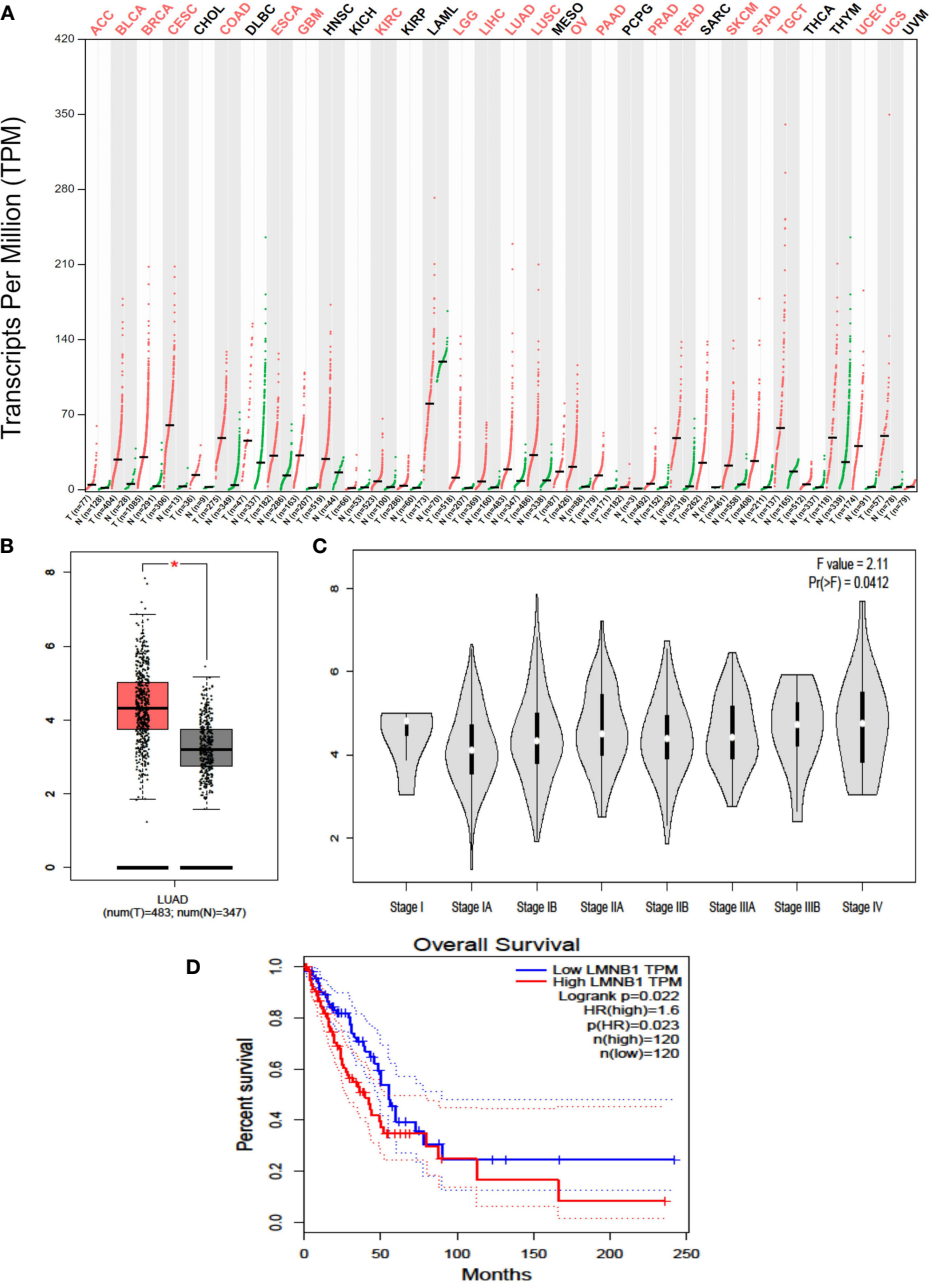
Highly expressed LMNB1 in LUAD cells is associated with tumor stage and overall survival. **(A)** The LMNB1 expression profile across all tumor samples and paired normal tissues was obtained on the GEPIA website (http://gepia.cancer-pku.cn/). **(B)** The expression of LMNB1 in the different pathological stages of LUAD patients from TCGA dataset by using bioinformatics’ analyses. *p<0.05. **(C)** The expression of LMNB1 at mRNA level in LUAD patients from TCGA and GTEx dataset by using bioinformatics’ analyses. **(D)** Overall survival analysis of LUAD patients from TCGA dataset (*P* = 0.022).

Firstly, the profile of LMNB1 expression across all cancer tissues and associated normal cells was retrieved from the GEPIA website (http://gepia.cancer-pku.cn/). LMNB1 is highly expressed in 21 different tumors, including LUAD (**Figure 1A**). The data on the transcription of LMNB1 mRNA in LUAD cancer cells and healthy cells was then obtained using bioinformatics approaches from the GTEx and TCGA databases. The analysis included 483 tumors tissues and 347 normal tissues, as shown in **Figure 1B**. The concentration of LMNB1 mRNA in target tissue was much higher in LUAD patients than in normal tissue, and the difference was found significant (P < 0.05). In addition, the production level of LMNB1 in LUAD patients increased with the clinical stages, as shown in **Figure 1C**. Next, we used bioinformatics to investigate the survival of LUAD patients from the database of TCGA. In total, 120 examples of LMNB1 high expression groups and LMNB1 low expression groups were obtained, respectively. Then, we compared the two groups of overall survival (OS). As shown in **Figure 1D**, there is a substantial specificity in OS between the two classes (P < 0.05).

### LMNB1 Knockdown Inhibits LUAD Cells Proliferation

**Figure 2:**
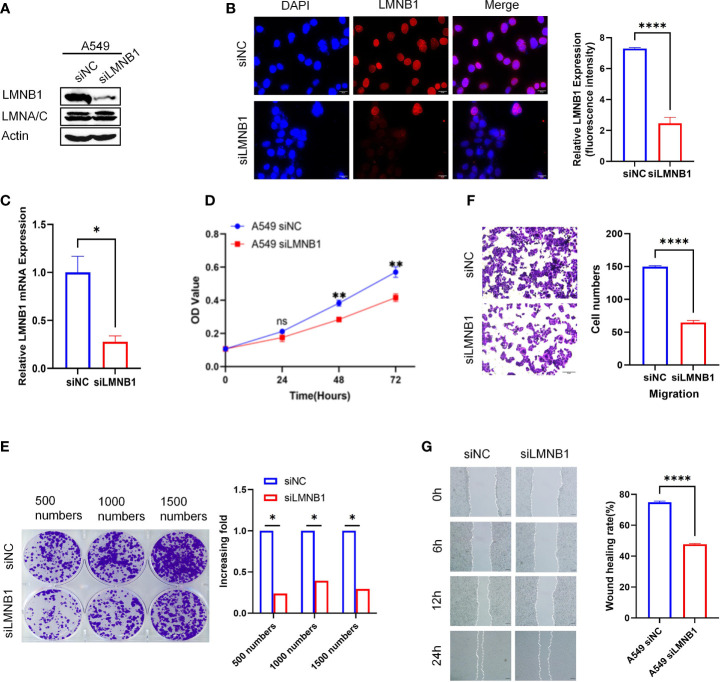
LMNB1 knockdown inhibits LUAD cells proliferation. **(A)** A549 cells were transfected with LMNB1 siRNAs, and WB detected the expression of protein LMNB1. **(B)** Immunofluorescence was used to detect the expression of LMNB1 (red fluorescence), and the relative fluorescence intensity was quantified with Image J (P < 0.05), Scale bar, 20 μm. **(C)** RT-qPCR was used to detect the mRNA expression of LMNB1. **(D)** CCK8 assay was conducted to measure the growth of A549 siLMNB1 cells. The absorbance value was measured at 490 nm (**P < 0.01). **(E)** Colony formation assay was conducted to measure the proliferation of A549 cell lines after knockdown of LMNB1. Histogram was used to analyze the colony numbers. **(F)** The Transwell assay was used to evaluate the migration ability of A549 cells after knocking down LMNB1. **(G)** Wound healing assay was conducted to measure cell migration and cell interactions of A549 siLMNB1. *p<0.05, ****p<0.0001. ns means no significance.

To further explore the effects of LMNB1 on LUAD cells, we used RNA interference in Lung adenocarcinoma cell A549 to knockdown the expression of LMNB1. WB, immunofluorescence, and qRT-PCR results showed that both the LMNB1 protein and RNA levels decreased (**Figures 2A–C**), while the expression of LMNA/C protein remained unchanged. The reduction of LMNB1 protein expression had no impact on the expression of LMNA/C. Then, cloning formation assay, wound healing assay Transwell assay, and CCK8 test were performed, and all results showed a slowdown in the growth of A549 cells knocked down LMNB1(**Figures 2D–G**). These results further demonstrate that knocking down LMNB1 inhibits LUAD cell proliferation.

### Knockdown of LMNB1 Increases Chromosome Accessibility *via* Decreases H3K9me3 Protein Expression

**Figure 3:**
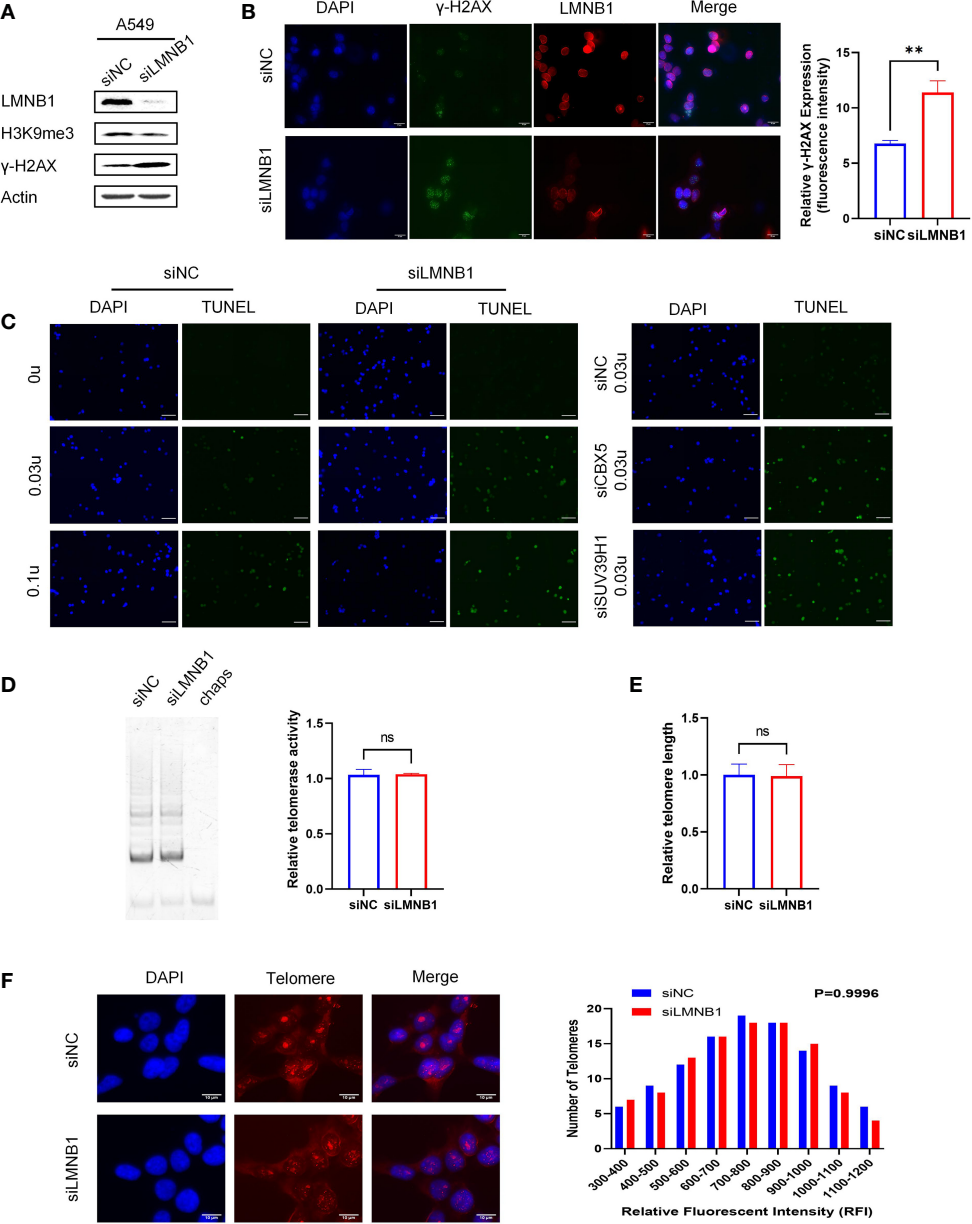
The knockdown of LMNB1 increases chromosome accessibility by decreasing H3K9me3 protein expression. **(A)** A549 cells were transfected with LMNB1 siRNAs, and WB detected the expression of protein LMNB1, H3K9me3, γ-H2AX, Actin. **(B)** Immunofluorescence was performed in A549 siLMNB1 cells to show γ-H2AX (green fluorescence) and LMNB1 (red fluorescence) protein expression, Scale bar, 20 μm. **(C)** DNase I-TUNEL assay was performed (DNase concentration used 0 U, 0.03 U, 0.1 U) to detect the difference in chromosome accessibility between A549 siLMNB1 cells and A549 siNC cells. A549 cells of siCBX5 and siSUV39H1 were used as positive controls, Scale bar, 50 μm. **(D)** The telomerase activity of knockdown of LMNB1 and NC were detected by TRAP in A549 cells. **(E)** The telomere length of A549 siLMNB1 and siNC were detected by qPCR. **(F)** The telomere length of A549 siLMNB1 and siNC were detected by Telo-fish. **p<0.01, ns means no significance.

Accompanied by a decrease of LMNB1 in A549 cells, to our surprise, the expression of chromosomal methylation protein H3K9me3 decreased. Instead, DNA damage-related protein markers γ-H2AX appeared to be elevated (**Figure 3A**), as confirmed by immunofluorescence experiments (**Figure 3B**). Immunofluorescence results showed that the cells with low LMNB1 expression showed more γ-H2AX expression. To verify that the loss of DNA methylation increases chromosome accessibility, we conducted the DNase I-TUNEL assay, with A549 cells knockdown CBX5 and SUV39H1 as positive controls, which has been proven to increase the chromosomes accessibility. Surprisingly, the LMNB1 knockdown group had a considerable increase in chromosome accessibility (**Figure 3C**). Telomeres are located in the heterogeneous chromatin region of chromosomes that are highly methylated. We tested telomere length and telomerase activity to verify whether the loss of methylation caused by knocking down LMNB1 affects telomeres. The results showed no change in them, which may be related to the brief absence of LMNB1 (**Figures 3D–F**). Perhaps, the ability of Telomerase to lengthen telomeres compensates for its shortening.

### The Absence of LMNB1 Can Induce LUAD Cells Senescence

**Figure 4:**
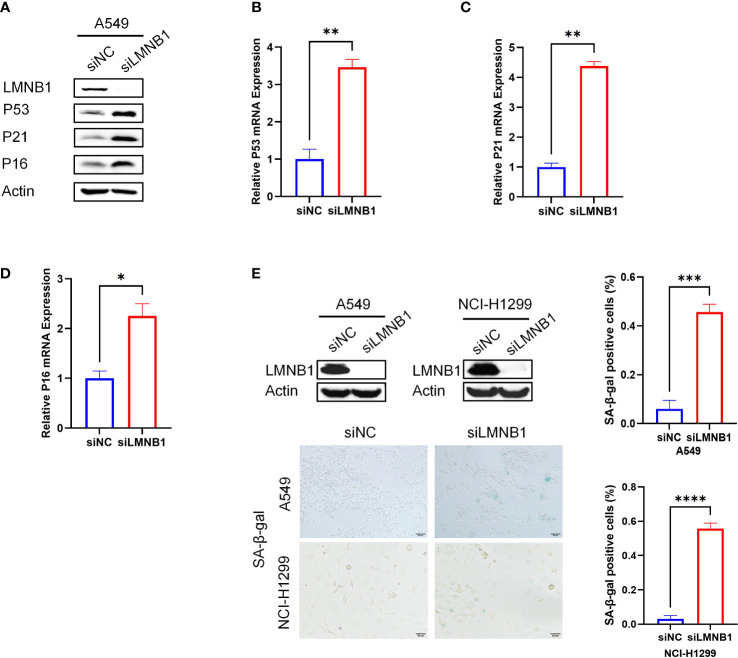
The absence of LMNB1 can induce LUAD cells senescence. **(A)** WB detected the expression of protein LMNB1, P53, P21, P16 and Actin in A549 siLMNB1 and siNC cells. **(B-D)** The expression of P53, P21 and P16 mRNA in A549 siLMNB1 cells and the control group were detected by RT-qPCR. **(E)** Proportion of senescent cells of A549 siLMNB1 cells and A549 siNC cells were detected by SA-β-Gal staining. The percentage of senescence positive cells in each group from five randomly chosen fields was calculated. *p<0.05. **p<0.01, ***p<0.001, ****p<0.0001.

To elucidate the mechanisms underlying the effect of LMNB1 on LUAD cell proliferation, we conducted WB experiments on A549 cells that knocked down LMNB1. The results showed that the proteins P53, P21 and P16 associated with cell senescence increased in the knockdown group (**Figure 4A**). Real-time PCR results also showed an increase in P53, P21 and P16 mRNA expression (**Figures 4B–D**). The lack of LMNB1 may inhibit the proliferation of LUAD by inducing cell senescence. The LMNB1 protein expression of A549 cells and NCI-1299 cells were knocked down using siRNAs. WB was used to detect the effect of knockdown. SA-β-gal assay further showed that cell senescence appeared in the LMNB1-knockdown cells (**Figure 4E**).

### Long-Term Loss of LMNB1 Can Cause Telomere Erosion

**Figure 5:**
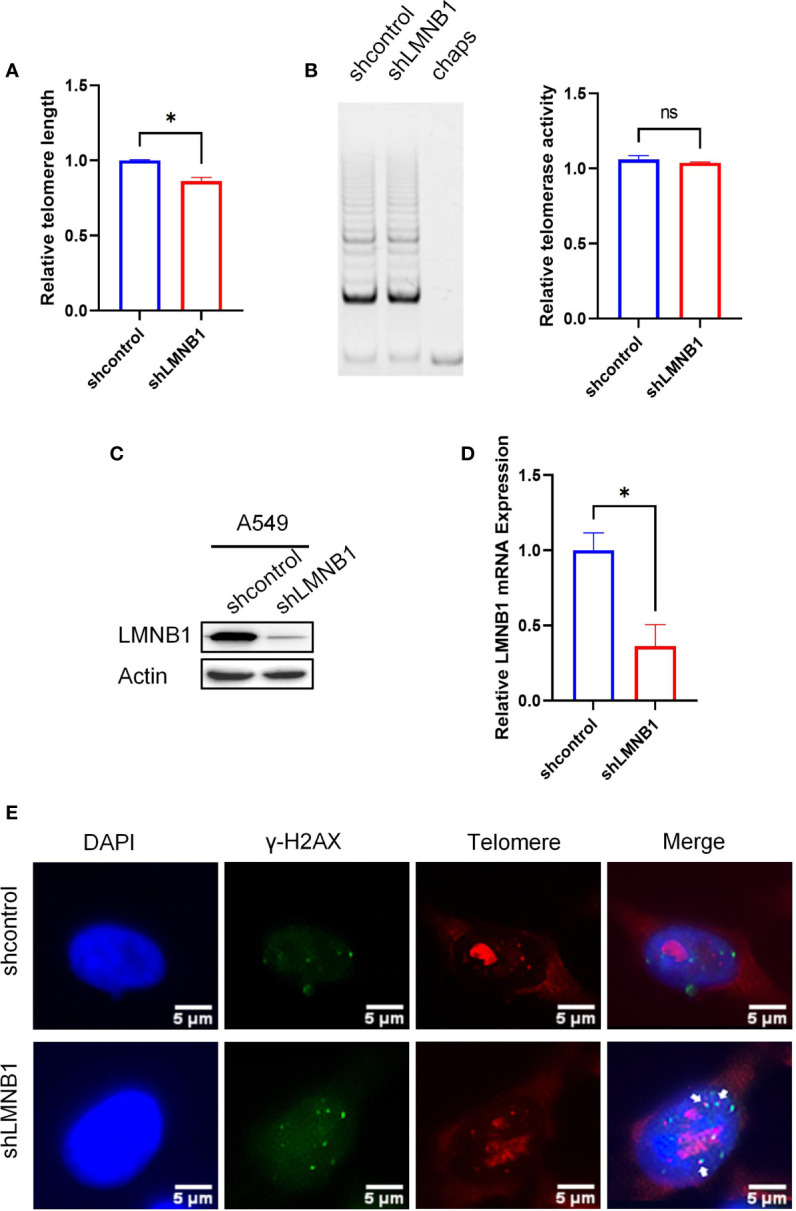
Long-term loss of LMNB1 can cause telomere erosion. **(A)** WB detected the expression of protein LMNB1, P53, P21, P16 and Actin in A549 siLMNB1 and siNC cells. **(B–D)** The expression of P53, P21 and P16 mRNA in A549 siLMNB1 cells and the control group were detected by RT-qPCR. **(E)** The proportion of senescent cells of A549(NCI-1299) siLMNB1 cells and A549 (NCI-1299) siNC cells were detected by SA-β-Gal staining. The percentage of senescence positive cells in each group from five randomly chosen fields were calculated. *p<0.05, ns means no significance.

Previous experiments have shown that the short-term reduction of LMNB1 increased chromosomal accessibility and DNA damage in cells, but made no difference to telomere length or telomerase activity. We wanted to further investigate the effect of long-term LMNB1 deficiency on telomeres, so that the A549 cells with long-term knockdown of LMNB1 (A549 shLMNB1) and control cells (A549 shcontrol) were tested for telomere length (**Figure 5A**). Surprisingly, the telomeres of cells were shortened. Western Blotting was performed to confirm the low concentration of LMNB1 (**Figure 5C**). qRT-PCR results showed that the LMNB1 RNA levels decreased (**Figure 5D**). In contrast, the long-term loss of LMNB1 caused the telomeres to become shorter but had no effect on telomerase activity (**Figure 5B**). It shows that the long-term lack of LMNB1 does not shorten telomeres by affecting the activity of telomerase. To explore the reasons for the shortening of telomeres, we performed immunofluorescence and telo-fish experiments. The results showed that the co-localization of γ-H2AX and telomeres increased (**Figure 5E**), and the increased DNA damage of telomeres might be an important reason for its shortening. To investigate whether telomere damage is caused by the changes in chromosome accessibility after the persistent deletion of LMNB1, ATAC-seq assay was performed. Sequencing connectors and the low-quality fragments of sequencing data were removed by trim. The rest of the high-quality reads are listed (**Figure S1A**). The number of reads obtained can reflect the chromosome accessibility of each group. Chromosome accessibility increased by 2% in the shLMNB1 group compared to the control (**Figure S1B**). To further show the changes that occurred in the telomere regions, that reads containing at least 6 tandem TTAGGG (or CCCTAA) were used to represent the accessibility of chromosomes in telomeres. To our surprise, the chromosomal accessibility in telomere regions increased significantly by nearly 26.7% compared to the control group (**Figure S1C**). Therefore, the cause of telomere shortening is that the increased chromosomal accessibility of the telomeric regions, and the accumulation of irreversible DNA damage occurs in the telomeres. The position of reads, as obtained in the shLMNB1 group, in the genome regions is shown in the **Figure S1D**. The results of GO enrichment analysis results showed that, there were significant differences in Biological Process (immune system process, cellular response to stimulus, and cell surface receptor signaling, etc.) between the shLMNB1 group and the control group (**Figure S1E**).

### Knockdown of LMNB1 Inhibits Tumor Growth in A549 Xenograft Models

**Figure 6:**
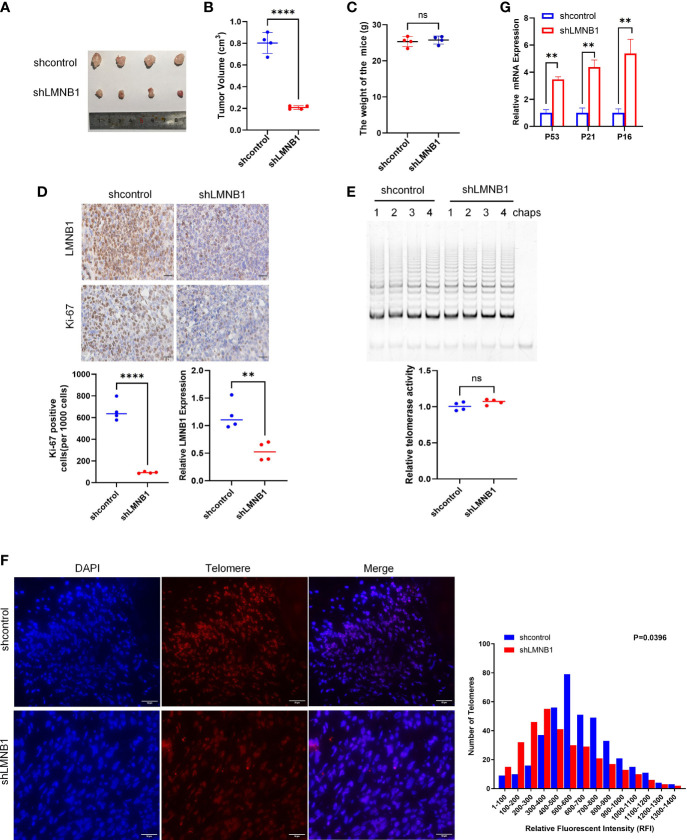
LMNB1 knockdown inhibits tumor growth in A549 xenograft models. **(A–C)** A549 cells stably expressing shcontrol or shLMNB1 were injected subcutaneously in nude mice (n = 4 for each group). Tumor value and mice bodyweight were measured 1 month later. **(D)** The expression of LMNB1 and Ki-67 in tumors of two groups were detected by immunohistochemistry. Scale bar, 30 μm. **(E)** Telomerase activity in tumors of each mice was detected by TRAP. **(F)** Telomere length was evaluated in paraffin embedded sections of tumor tissue using Q-FISH. The Image J software was used to analyze the telomere length of tumor sections, as indicated by fluorescence intensity. Scale bar, 50 μm. **(G)** The expression of P53, P21 and P16 mRNA in tumors were detected by RT-qPCR. **p<0.01, ****p<0.0001, ns means no significance.

To learn more about the involvement of LMNB1 in tumorigenesis and proliferation *in vivo*, we injected shcontrol A549 cells and shLMNB1 A549 cells subcutaneously, respectively, for establishing a xenograft tumor model in tumor-bearing mice. After 7 weeks, the weights of the mice and tumors were measured separately after the mice were euthanized. The tumors volume of the shLMNB1 group was substantially decreased compared to the shcontrol group, as illustrated in **Figures 6A–C**. However, there was no difference in weight between the two groups. It indicates that the deletion of LMNB1 could inhibit the growth of cells *in vivo*. Furthermore, IHC established that after the knockdown of LMNB1, the protein levels of Ki-67 and LMNB1 in tumor tissues were considerably down-regulated (**Figure 6D**). Consistent with the results of cell experiments, Telomerase activity did not decrease considerably following LMNB1 knockdown as it did *in vitro* (**Figure 6E**). Compared with the control group, the shLMNB1 group had shorter telomeres (**Figure 6F**), and RT-qPCR outcomes show that the mRNA levels of P53, P21 and P16 proteins all increased (**Figure 6G**), which suggests that the lack of LMNB1 can also cause telomere shortening and induce cell senescence *in vivo*.

## Discussion

There has been a significant advancement in lung cancer early detection and minimally invasive treatment in recent years. The development of targeted molecular therapies (such as gefitinib) has opened up new ideas for treating NSCLC. Recently, new biological targets such as ALK, ROS1, and PD-L1 have been evaluated in targeted clinical therapy (18–21). However, clinically, there is still a lack of effective treatment methods. According to statistics, the life expectancy for NSCLC remains low, and the prognosis of LUAD patients is very poor (22). Therefore, there is an urgent need to have a more profound knowledge of the biological mechanism of the incidence and progression of LUAD, in addition to the discovery and study of new biological markers and therapeutic targets to achieve early diagnosis, precise treatment, and improve patient prognosis.

Lung cancer is the most frequently diagnosed tumor globally, with suggestions for an increasing trend in younger-onset age (23). Because of the late detection of lung cancer, early metastasis, and high fatality rate, the overall prognosis of patients is poor (2, 24). The incidence of lung cancer in men ranks first among all types of tumors and lung cancer is also first in terms of cancer deaths; in women, it is the third most significant cancer and the second leading cause of tumor mortality (25). It is worth noting that lung cancer results from multiple genes and multiple factors. So far, the serum tumor markers used in early lung cancer diagnosis and recurrence monitoring include CEA, secreted protein Dickopf-1 (DKK1), etc (26). Adenocarcinoma (LUAD) is now the most frequent lung cancer, accounting for more than half of all cases. As a result, enhancing the early diagnosis rate of LUAD, discovering novel prognostic indicators, and discovering new targets for anti-tumor therapy has been the focus of lung therapeutic strategies.

The lamins, recognized as intermediate filament protein (IF) by sequence similarity, were the first proteins located in the nuclear layer to be biochemically identified (27–32). Lamins, located inside the nuclear membrane, provided a platform for combining protein and chromatin and impacted mechanical stability. Besides, they involve many nuclear functions such as high-order genome organization, chromatin regulation, transcription, DNA replication, and DNA repair.

Nuclear lamins can be divided into two classes- Type A, and Type B. Type A and Type B lamins are different in protein structure, expression, localization mode, and biochemistry (33). LMNB1 was discovered to display a crucial role in senescence and proliferation of the cell, DNA replication and gene expression, chromosomal dispersion and aggregation, DNA fragmentation, and telomere stability, in addition to preserving the integrity and shape of the cell nucleus. It has been proven that abnormal LMNB1 expression is linked to neurological diseases and tumors (16, 34–37). Given that LMNB1 has different and complex biological functions in other tumor cells, in-depth research will lay a foundation for studying the pathological mechanisms of various diseases and exploring innovative biomarkers and targets for cancer therapy.

The occurrence and development of LUAD is closely related to lamins. The down-regulation of Lamin A/C expression confers higher variability in the nuclear morphology of cancer cells and ultimately promotes tumor metastasis (38, 39). The study by Pajerowski et al. (40) showed that: the lack of Lamin A/C enhanced the nuclear deformability in lung adenocarcinoma cell line A549. However, LMNB1 is required for DNA replication, gene expression, cell proliferation and senescence, chromosome dispersion and aggregation, DNA fragmentation, and telomere homeostasis (41). It has been established that aberrant LMNB1 expression is linked to the development of neurological disorders and tumors (15, 42, 43). Furthermore, researchers have found that LaminB1 is highly expressed in lung adenocarcinoma and stimulates proliferation in lung tumor cells *via* the AKT pathways (44).

Our research established that silencing LMNB1 inhibits the growth of LUAD cells by inducing cell senescence. Through multiple experiments, we found that the loss of LMNB1 caused a decrease in the expression of H3k9me3 protein, thereby increasing the accessibility of chromosomes, and the DNA damage standard γ-H2AX protein was detected. Hayflick and Moorhead were the first to describe cell aging as a progressive and irreversible loss of the proliferation potential of human cells (45). This phenomenon is characterized by the loss of replication ability, as well as by several variations of cell morphology, gene expression, metabolism, epigenetics, etc (46). Thus far, telomeres shortening optimally accounts for replicable aging, which is a repeating area of DNA at the end of chromosomes and presents a high methylation state. It has been reported that telomere regions are shortened as cells divide, which is correlated with the induction of cell senescence (47). However, tumor cells can express telomerase, which resists replication aging by maintaining telomere length (48). Under normal circumstances, DNA damage caused by daily cell damage can be repaired effectively and quickly. However, studies have shown that damage to telomeres is difficult to repair *in vivo* and *in vitro* (49). Persistent telomere DNA damage under various causes is a feature of replication, stress and carcinogenic gene-induced aging, so unrepaired telomeres are considered to provide a long-lasting source of DDR signals, which is of critical significance to establishing cell senescence (50). To the best of our knowledge, our study is the first to show that continual deletion of LMNB1 in LUAD cells induces DNA damage to telomeres and shortens telomeres. Therefore, we speculate that targeting LMNB1 may present a new avenue for LUAD treatment.

## Data Availability Statement

The original contributions presented in the study are included in the article/**Supplementary Material**. Further inquiries can be directed to the corresponding authors.

## Ethics Statement

The animal study was reviewed and approved by Institutional Animal Care and Use Committee of the Beijing Institute of Biotechnology.

## Author Contributions

JL, ZS and YC performed experiments, performed analysis and wrote manuscript. LQ, FW and YL performed analysis. XL, JL and ND supervised study, provided reagents and software and edited manuscript. All authors contributed to the article and approved the submitted version.

## Funding

National Natural Science Foundation of China (61975239)

## Conflict of Interest

The authors declare that the research was conducted in the absence of any commercial or financial relationships that could be construed as a potential conflict of interest.

## Publisher’s Note

All claims expressed in this article are solely those of the authors and do not necessarily represent those of their affiliated organizations, or those of the publisher, the editors and the reviewers. Any product that may be evaluated in this article, or claim that may be made by its manufacturer, is not guaranteed or endorsed by the publisher.

## Supplementary Material

Supplementary Figure 1 ATAC-seq. **(A)** The number and characteristics of the reads as obtained by ATAC-seq assay. **(B)** The chromosomal accessibility in each group was reflected in the number of reads. **(C)** The reads containing at least 6 tandem TTAGGG (or CCCTAA) were used to represent the chromosomes accessibility in telomeres. **(D)** The distribution of reads on chromosomes in the shLMNB1 group. **(E)** The most enriched of GO terms were displayed. (Diff_peak_shLMNB1_vs_shcontrol).
