# Supplementary figures and images for: Knockdown of LMNB1 Inhibits the Proliferation of Lung Adenocarcinoma Cells by Inducing DNA Damage and Cell Senescence
Source: Front Oncol. 2022 May 31;12:913740. doi: 10.3389/fonc.2022.913740 (PMC9194513; doi:10.3389/fonc.2022.913740)

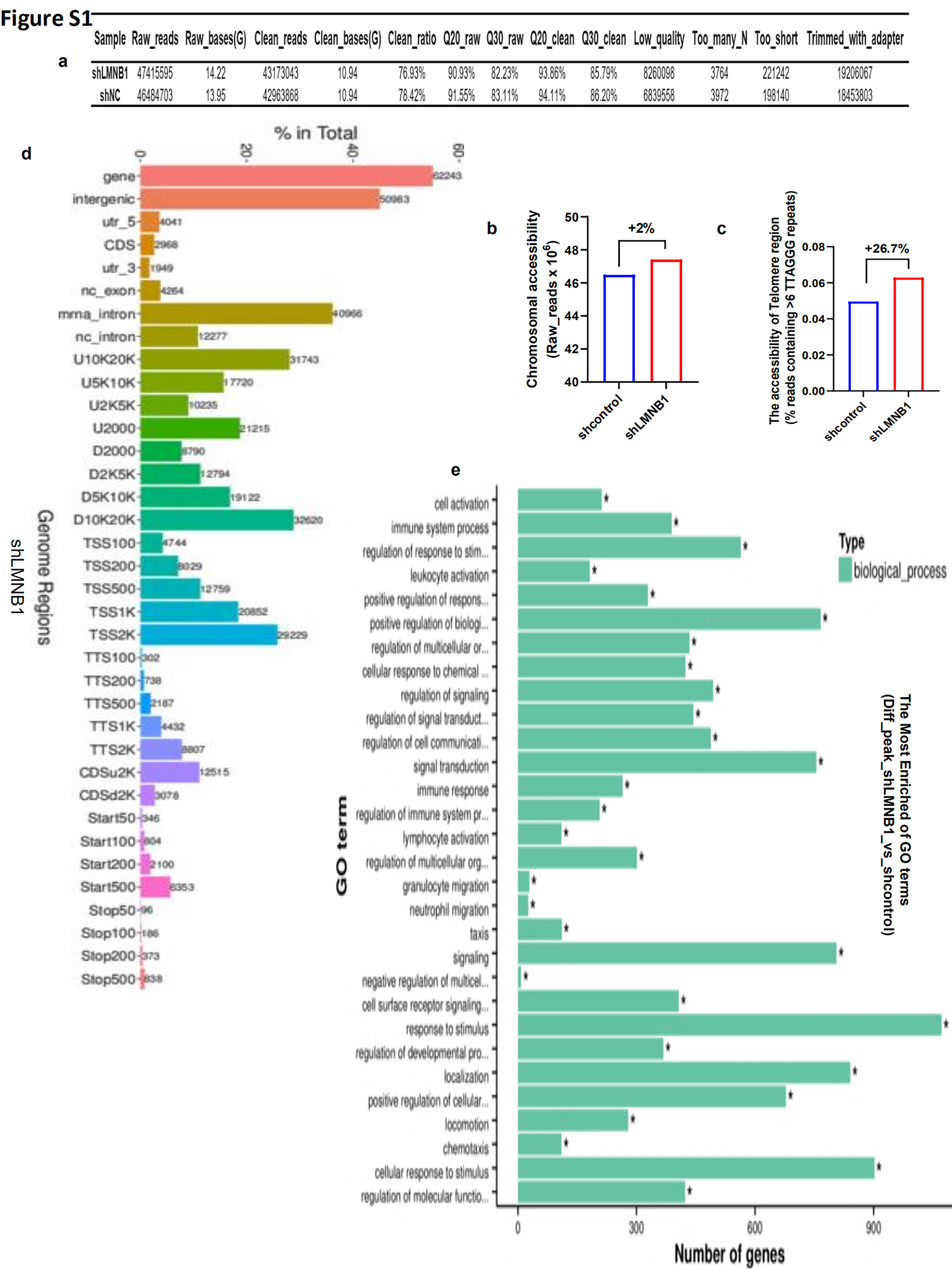

Supplement: Supplementary Figure 1 — ATAC-seq. (A) The number and characteristics of the reads as obtained by ATAC-seq assay. (B) The chromosomal accessibility in each group was reflected in the number of reads. (C) The reads containing at least 6 tandem TTAGGG (or CCCTAA) were used to represent the chromosomes accessibility in telomeres. (D) The distribution of reads on chromosomes in the shLMNB1 group. (E) The most enriched of GO terms were displayed. (Diff_peak_shLMNB1_vs_shcontrol). [file Image_1.tif]
